# Interleukin-37 suppresses the cytotoxicity of hepatitis B virus peptides-induced CD8+ T cells in patients with acute hepatitis B

**DOI:** 10.17305/bjbms.2022.8260

**Published:** 2023-05-01

**Authors:** Qian Liu, Qiang Zhou, Mingrui Wang, Bo Pang

**Affiliations:** 1 Center for Reproductive Medicine, Center for Prenatal Diagnosis, The First Hospital of Jilin University, Changchun, Jilin Province, China; 2 Department of Hepatology, The First Hospital of Jilin University, Changchun, Jilin Province, China; 3 Department of Obstetrics and Gynecology, Affiliated Hospital of Guangdong Medical University, Zhanjiang, Guangdong Province, China; 4 Department of Cardiology, The First Hospital of Jilin University, Changchun, Jilin Province, China

**Keywords:** Hepatitis B virus (HBV), acute infection, CD8+ T cells, cytotoxicity, interleukin-37 (IL-37)

## Abstract

Interleukin-37 (IL-37) is a newly identified anti-inflammatory cytokine, owning immunosuppressive activity in infectious diseases. The aim of this study was to investigate the regulatory function of IL-37 on CD8+ T cells during hepatitis B virus (HBV) infection. Eighteen acute hepatitis B (AHB) patients, 39 chronic hepatitis B (CHB) patients, and 20 controls were enrolled. IL-37 concentration was measured by enzyme-linked immunosorbent assay. IL-37 receptor subunits expressions on CD8+ T cells were assessed by flow cytometry. Purified CD8+ T cells were stimulated with HBV peptides and recombinant IL-37. Perforin and granzyme B secretion was investigated by ELISPOT. Programmed death-1 (PD-1) and cytotoxic T-lymphocyte-associated protein-4 (CTLA-4) mRNA expressions were semi-quantified by real-time PCR. CD8+ T cell cytotoxicity was assessed in direct contact and indirect contact coculture with HepG2.2.15 cells. Plasma IL-37 level was down-regulated and negatively correlated with aminotransferase levels in AHB patients. There were no significant differences of IL-37 receptor subunits among AHB patients, CHB patients, and controls. Exogenous IL-37 stimulation suppressed HBV peptides-induced perforin and granzyme B secretion by CD8+ T cells in AHB patients, but not in CHB patients. Exogenous IL-37 stimulation did not affect proinflammatory cytokines secretion as well as PD-1/CTLA-4 mRNA expressions in CD8+ T cells in AHB and CHB patients. Exogenous IL-37 stimulation dampened HBV peptide-induced CD8+ T cell cytotoxicity in a cell-to-cell contact manner. The current data indicated that acute HBV infection might induce down-regulation of IL-37, which might be associated with enhanced CD8+ T cell cytotoxicity and liver damage.

## Introduction

Hepatitis B virus (HBV) infection is not directly cytopathic for hepatocytes, and the clinical outcome of HBV infection is the result of complicated interaction between virus and host immune response [1]. Infection with HBV in adults frequently results in a self-limiting and acute hepatitis, which confers protective immunity and causes no further disease. The multispecific T cell response with proinflammatory cytokine production is not only important for controlling of the infection but also contributes to severe liver injury [2, 3]. In 10% of adults and 90% of children, HBV always leads to chronic and persistent infection. Cellular immune responses are weak or undetectable, which reflects the state of relative collapse of HBV-specific adaptive immunity [4, 5]. However, the precise mechanism underlying the differential immune response in acute hepatitis B (AHB) and chronic hepatitis B (CHB) patients remains not completely understood.

Cytokine balance is an essential immune characteristic in development and progression of HBV infection. An increasing numbers of cytokines are involved in the initiation and modulation of immune response to HBV, being the primary causes of liver inflammation and effective antiviral agents [6]. Interleukin-37 (IL-37) is a newly identified cytokine, belongs to IL-1 family [7]. IL-37 receptor contains two different subunits, including IL-18 receptor α chain (IL-18Rα) and single immunoglobin IL-1 receptor-related protein (SIGIRR). Signaling through IL-37/IL-37 receptor leads to trigger multiple intracellular switches, resulting in down-regulation of proinflammatory genes and suppression of cytokine production [8]. Thus, IL-37 owns potent immunosuppressive activity against innate and acquired immune responses through inhibition of inflammatory mediators in cancers, autoimmune diseases, and infectious diseases [7]. IL-37 suppressed Th17 response and augmented regulatory T cell function, leading to alleviation of Coxsackievirus B3-induced viral myocarditis [9] and hand, foot, and mouth disease [10]. IL-37 inhibited the development of schistosomiasis and ameliorated Schistosoma japonicum-induced liver granuloma by promoting polarization of macrophages to M2 phenotype [11]. Neutralization of IL-37 enhanced frequencies of CD4+ and CD8+ T cell subsets with parasite antigen stimulation during Strongyloides stercoralis infection [12]. Importantly, IL-37-mediated CD8+ T cell dysfunction through SIGIRR, leading to dampening of protective cytotoxic T cell-induced immunity in colitis-associated colorectal cancer [13].

**Table 1:** Clinical and demographic characteristic of enrolled subjects

	**Acute hepatitis B (AHB)**	**Chronic hepatitis B (CHB)**	**Controls**
Case (*n*)	18	39	20
Sex (male/female)	12/6	27/12	13/7
Age (years)	36.06 ± 11.60	30.41 ± 8.60	30.90 ± 10.90
ALT (IU/L)	489 (286, 1101)	165 (94, 263)	19 (14, 29)
AST (IU/L)	415 (213, 846)	101 (52, 168)	27 (20, 33)
HBsAg positive (*n*)	18	39	Not available
HBeAg positive (*n*)	9	35	Not available
Anti-HBe positive (*n*)	8	4	Not available
Anti-HBc IgM titer	26.56 (14.54, 33.21)	Not available	Not available
HBV DNA (log_10_IU/L)	4.83 ± 1.42	7.57 ± 1.27	Not available

ALT: Alanine aminotransferase; AST: Aspartate aminotransferase; HBsAg: Hepatitis B surface antigen; HBeAg; hepatitis B e antigen; anti-HBc: Anti-hepatitis B core antibody; HBV: Hepatitis B virus.

Serum IL-37 level was shown to be associated with liver damage and played a significant role in the immune response of CHB patients with hepatitis B e antigen (HBeAg) seroconversion [14]. However, IL-37 expression profile in AHB patients and the regulatory function of IL-37 to CD8+ T cells during HBV infection was not fully elucidated. Due to the immunosuppressive property of IL-37, we hypothesized that IL-37 contributed to inhibition of CD8+ T cell activity in patients with HBV infection. To test this possibility, we investigated IL-37 and IL-37 receptor expression in HBV-infected patients and assessed the immunomodulatory function of recombinant human IL-37 stimulation to HBV peptides-induced CD8+ T cells using in vitro cell culture system.

## Materials and methods

### Enrolled subjects

Eighteen AHB patients and 39 CHB patients were enrolled in this study. Diagnosis of AHB was made according to the following criteria: (1) Denying the history of chronic HBV infection; (2) Positive for Hepatitis B surface antigen (HBsAg) and anti-hepatitis B core antibody (anti-HBc) IgM; (3) Positive for HBV DNA; (4) Severe liver injury with more than 5-fold elevation of serum alanine aminotransferase (ALT). Diagnosis of CHB was made according to the following criteria: (1) Positive for HBsAg for more than six months; (2) Positive for HBV DNA; (3) Serum ALT was higher than upper limits of normal. All patients were treatment-naïve for antiviral or immunomodulatory therapies. No patients were afflicted with other chronic viral infections, autoimmune disorders, or end-stage liver diseases. For controls, 20 age- and sex-matched healthy individuals were also included. All enrolled subjects are Chinese. The clinical and demographic characteristics of all enrolled subjects are shown in Table 1.

### Isolation of plasma and peripheral blood mononuclear cells

Twenty milliliters (ml) of ethylene diamine tetraacetie acid-anticoagulant peripheral blood samples were collected from each enrolled subjects. Plasma samples were harvested by centrifugation at 3000×*g* for 10 min. Peripheral blood mononuclear cells (PBMCs) were isolated using Ficoll-Hypaque (Solarbio, Beijing. China) density gradient centrifugation. Approximate 2 × 107 of PBMCs could be harvested from 20 ml of peripheral blood.

### Flow cytometry analysis

PBMCs were stained with fluorescein isothiocyanate (FITC) mouse anti-human CD8 (Clone RPA-T8) (BD Pharmingen, San Jose, CA, USA), phycoerythrin (PE) mouse anti-human CD218α (IL-18Rα) (Clone H44) (BD Pharmingen, San Jose, CA, USA), and human SIGIRR allophycocyanin (APC) conjugated antibody (Clone #162201) (R&D Systems, Minneapolis, MN, USA) in the dark for 30 min at 4 ^∘^C. The isotype control was used for separation of positive and negative cells of IL-18Rα and SIGIRR. Samples were analyzed using FACS Calibur analyzer (BD Biosciences, San Jose, CA, USA). Acquisitions were performed using CellQuest Pro software (BD Biosciences, San Jose, CA, USA). Analyses were performed using FlowJo V10 software (TreeStar, Ashland, OR, USA).

### Purification of CD8+ T cells

CD8+ T cells were purified using Human CD8+ T Cells Isolation Kit (Miltenyi, Bergisch Gladbach, Germany) following manufacturer’s instructions. The purification rate was approximately 20%~30%. The purity of enriched CD8+ T cells was more than 95% following flow cytometry determination.

### Enzyme-linked immunospot assay

Purified CD8+ T cells from AHB and CHB patients were stimulated with HBV C genotype core peptide pool (15 amino acids of each peptide with 5 amino acids overlapping) (final concentration: 2.5 µg/ml) in the presence or absence of recombinant human IL-37 (Peprotech, Rocky Hill, NY, USA; final concentration: 0.1 ng/ml) for 96 h [15, 16]. Perforin and granzyme B secretion by CD8+ T cells were measured using Human Perforin enzyme-linked immunospot assay (ELISPOT) Kit (Abcam, Cambridge, MA, USA) and Human Granzyme B ELISPOT Kit (Abcam, Cambridge, MA, USA), respectively. The results were shown as numbers of spot-forming cells (SFCs).

### Real-time polymerase chain reaction

Real-time polymerase chain reaction (PCR) was performed as previously described [17]. Total RNA was purified from cultured CD8+ T cells using RNeasy Mini Kit (Qiagen, Hilden, Germany) following manufacturer’s instructions. First-strand cDNA was synthesized with random hexamers using PrimeScript RT Master Mix (TaKaRa, Beijing, China). Real-time PCR was performed using SYBR Premix Ex Taq (TaKaRa). mRNA relative expressions of programmed death-1 (PD-1) and cytotoxic T-lymphocyte-associated protein-4 (CTLA-4) were quantified by 2-ΔΔCT method using Applied Biosystems 7500 System Sequence Detection software (Applied Biosystems, Foster, CA, USA). The primer sequences were cited from the previous studies [18].

### Cell culture

Purified CD8+ T cells from eight HLA-A*02 positive AHB patients were stimulated with recombinant human IL-37 (0.1 ng/ml) for 24 h. After washed twice to remove exogenous IL-37, enriched CD8+ T cells were co-cultured in direct or indirect contact with HepG2.2.15 cells, which were also HLA-A*02 positive [19]. In direct contact coculture system, 1×104 of CD8+ T cells and 5×104 of HepG2.2.15 cells were directly mixed, and cultured in a 24-well plate for 48 h in the presence of recombinant HBsAg (AbD Serotec, Oxford, United Kingdom; final concentration: 10 µg/ml) and HBc18-27 peptide (sequence: FLPSDFFPSV; final concentration: 10 µg/ml). In indirect contact coculture system, a Transwell plate (Corning, Corning, NY, USA) was used. 5×104 of HepG2 cells were seeded into lower chamber of the plate, while 1×104 of CD8+ T cells were added into upper chamber. The effector and target cells were separated by a 0.4-µm membrane. Recombinant HBsAg and HBc18-27 peptide was also added into upper chamber for maintenance of T cell activity. Supernatants were harvested 48 h post coculture.

### Cytotoxic assay

The cytotoxicty of CD8+ T cells to HepG2.2.15 cells was calculated by measuring lactate dehydrogenase (LDH) level in the supernatants. Briefly, LDH level in the supernatants of HepG2.2.15 cells was defined as “low-level control.” LDH level in the supernatants of Triton X-100 treated HepG2.2.15 cells was defined as “high-level control.” The percentage of CD8^+^ T cell-induced HepG2.2.15 cell death was calculated by the following formula: (high level control - LDH level)/(high level control - low level control)×100%.

### Enzyme-linked immunosorbent assay

IL-37 and soluble SIGIRR (sSIGIRR) level in the plasma, as well as interferon-γ (IFN-γ), tumor necrosis factor-α (TNF-α), perforin, and granzyme B levels in the supernatants were measured using commercial enzyme-linked immunosorbent assay (ELISA) kits (Cusabio, Wuhan, Hubei Province, China; Invitrogen, Carlsbad, CA, USA) following manufacturers’ instructions.

### Ethical statement

The study protocol was approved by the Ethics Committee of The First Hospital of Jilin University (No. 2015-015). Written informed consent was obtained from each subject.

### Statistical analysis

All data were analyzed using SPSS 23.0 for Windows software (Chicago, IL, USA). Shapiro–Wilk test was used for normal distribution assay. Data following normal distribution were presented as mean ± standard deviation. The one-way analysis of variance (ANOVA) and Student–Newman–Keuls-q (SNK-q) test was used when more than two groups were being compared. The Student’s *t* test was used when two groups were being compared. The paired *t* test was used when two groups (prior to and post stimulation) were being compared. Data following skewed distribution were presented as median (Q1, Q3). Pearson or Spearman correlation test was used for correlation analysis. All tests were two tailed, and *P* values of less than 0.05 were considered to indicate significant differences.

## Results

### Plasma IL-37 level was down-regulated and negatively correlated with aminotransferase levels in AHB patients

**Figure 1.**
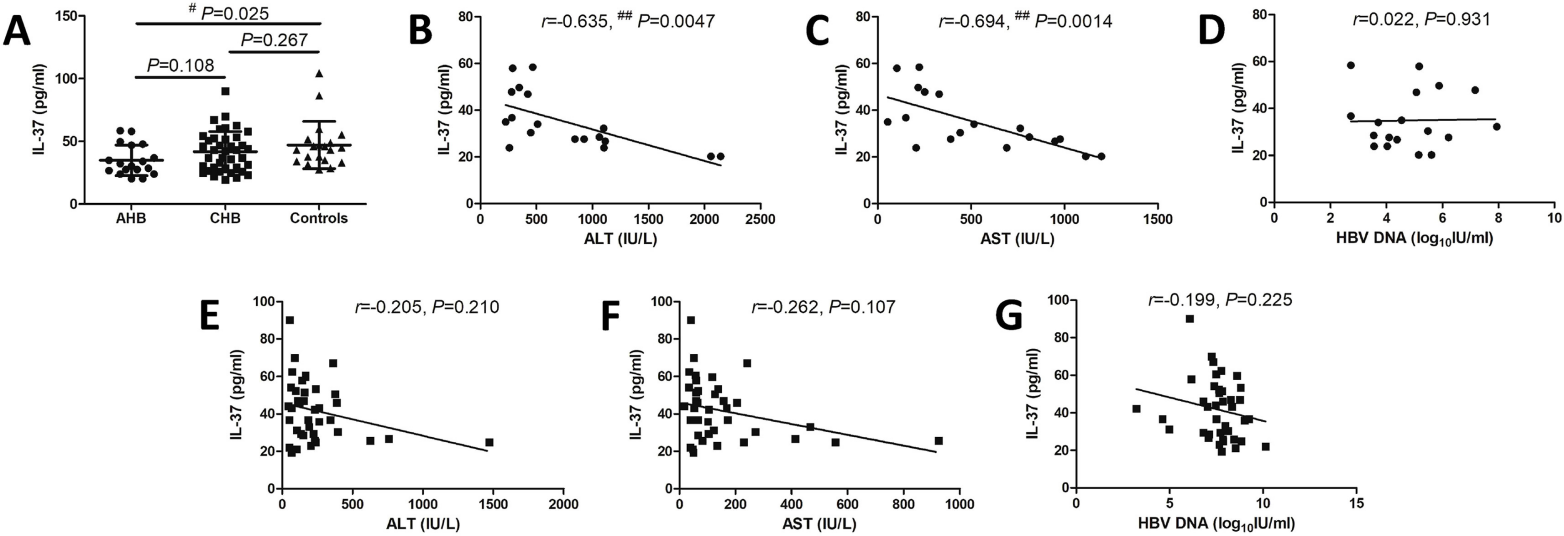
**Plasma IL-37 level in acute hepatitis B (AHB) patients, chronic hepatitis B (CHB) patients, and controls.** Plasma IL-37 level was measured by enzyme-linked immunosorbent assay in all enrolled subjects, including AHB patients (*n* ═ 18), CHB patients (*n* ═ 39), and controls (*n* ═ 20). (A) Plasma IL-37 level was compared among AHB patients, CHB patients, and controls. The one-way analysis of variance and Student–Newman–Keuls-q test was used for comparison; (B) The correlation between plasma IL-37 level and alanine aminotransferase (ALT) level in AHB patients was analyzed by Spearman correlation test; (C) The correlation between plasma IL-37 level and aspartate aminotransferase (AST) level in AHB patients was analyzed by Spearman correlation test; (D) The correlation between plasma IL-37 level and HBV DNA level in AHB patients was analyzed by Pearson correlation test; (E) The correlation between plasma IL-37 level and ALT level in CHB patients was analyzed by Spearman correlation test; (F) The correlation between plasma IL-37 level and AST level in CHB patients was analyzed by Spearman correlation test; (G) The correlation between plasma IL-37 level and HBV DNA level in CHB patients was analyzed by Pearson correlation test.IL-37: Interleukin-37; HBV: Hepatitis B virus; ^#^*P* < 0.05; ^##^*P* < 0.005.

IL-37 level in the plasma of all enrolled subjects were firstly screened. Plasma IL-37 level in AHB patients (34.88 ± 12.21 pg/ml) was notably reduced when compared with controls (47.07 ± 18.88 pg/ml; SNK-q test, *P* ═ 0.025, Figure 1A). However, there was no remarkable difference of plasma IL-37 level between CHB patients (41.82 ± 15.99 pg/ml) and controls (SNK-q test, *P* ═ 0.267, Figure 1A). Plasma IL-37 level was negatively correlated with alanine aminotransferase (ALT) (Spearman correlation test, *r* ═ −0.635, *P* ═ 0.0047, Figure 1B) and aspartate aminotransferase (AST) (Spearman correlation test, *r* ═ −0.694, *P* ═ 0.0014, Figure 1C) in AHB patients. However, there were no significant correlations between plasma IL-37 level and aminotransferases in CHB patients (Spearman correlation tests, *P* > 0.05, Figure 1E and 1F). IL-37 level in the plasma did not correlate with HBV DNA level in either AHB patients (Pearson correlation test, *r* ═ 0.022, *P* ═ 0.931, Figure 1D) or CHB patients (Pearson correlation test, *r* ═ −0.199, *P* ═ 0.225, Figure 1G).

### There were no significant differences of IL-37 receptor levels between AHB patients, CHB patients, and controls

**Figure 2.**
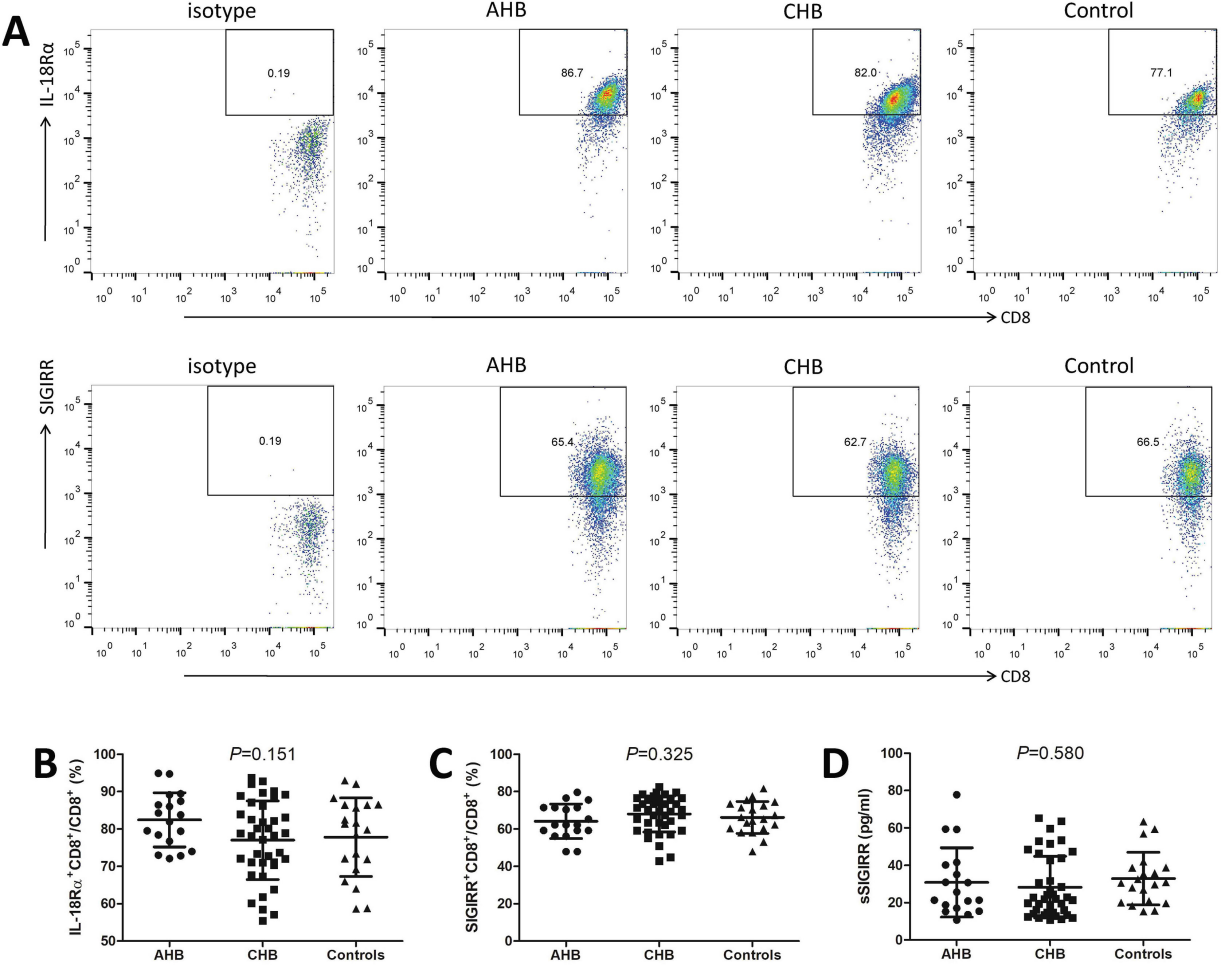
**Expressions of IL-37 receptor subunits on CD8+ T cells and soluble single immunoglobin IL-1 receptor-related protein (SIGIRR) in acute hepatitis B (AHB) patients, chronic hepatitis B (CHB) patients, and controls.** Peripheral blood mononuclear cells (PBMCs) were isolated from all enrolled subjects, including AHB patients (*n* ═ 18), CHB patients (*n* ═ 39), and controls (*n* ═ 20). PBMCs were stained with anti-CD8-FITC, anti-IL-18 receptor α chain (IL-18Rα)-PE, and anti-SIGIRR-APC. Cells were analyzed by flow cytometry. (A) The representative flow dots for IL-18Rα and SIGIRR expression on CD8+ T cells in AHB patients, CHB patients, and controls were shown. The isotype control was used for separation of positive and negative cells of IL-18Rα and SIGIRR; The percentage of (B) IL-18Rα+CD8+ cells and (C) SIGIRR+CD8+ cells within CD8+ T cells were compared among AHB patients, CHB patients, and controls; (D) Plasma soluble SIGIRR (sSIGIRR) level was measured by enzyme-linked immunosorbent assay, and was compared among AHB patients, CHB patients, and controls. The one-way analysis of variance was used for comparison.IL-37: Interleukin-37; FITC: Fluorescein isothiocyanate; PE: Phycoerythrin; APC: Allophycocyanin.

The expressions of IL-37 receptor subunits, IL-18Rα and SIGIRR, on CD8+ T cells of all enrolled subjects were then investigated. The representative flow dots analyses for IL-18Rα and SIGIRR expression on CD8+ T cells in AHB patients, CHB patients, and controls were shown in Figure 2A. The percentage of IL-18Rα-positive cells within CD8+ T cells in AHB patients (82.46 ± 7.22 %) was slightly higher than in CHB patients (77.01 ± 10.53 %) and controls (77.81 ± 10.52 %), but this difference were failed to achieve statistical significance (One-way ANOVA, *P* ═ 0.151, Figure 2B). There was no remarkable difference of SIGIRR+CD8+ cell percentage among AHB patients, CHB patients, and controls (One-way ANOVA, *P* ═ 0.325, Figure 2C). Plasma sSIGIRR level was also comparable among AHB patients, CHB patients, and controls (One-way ANOVA, *P* ═ 0.580, Figure 2D).

### Exogenous IL-37 stimulation suppressed HBV peptides-induced perforin and granzyme B secretion by CD8+ T cells in AHB and CHB patients

**Figure 3.**
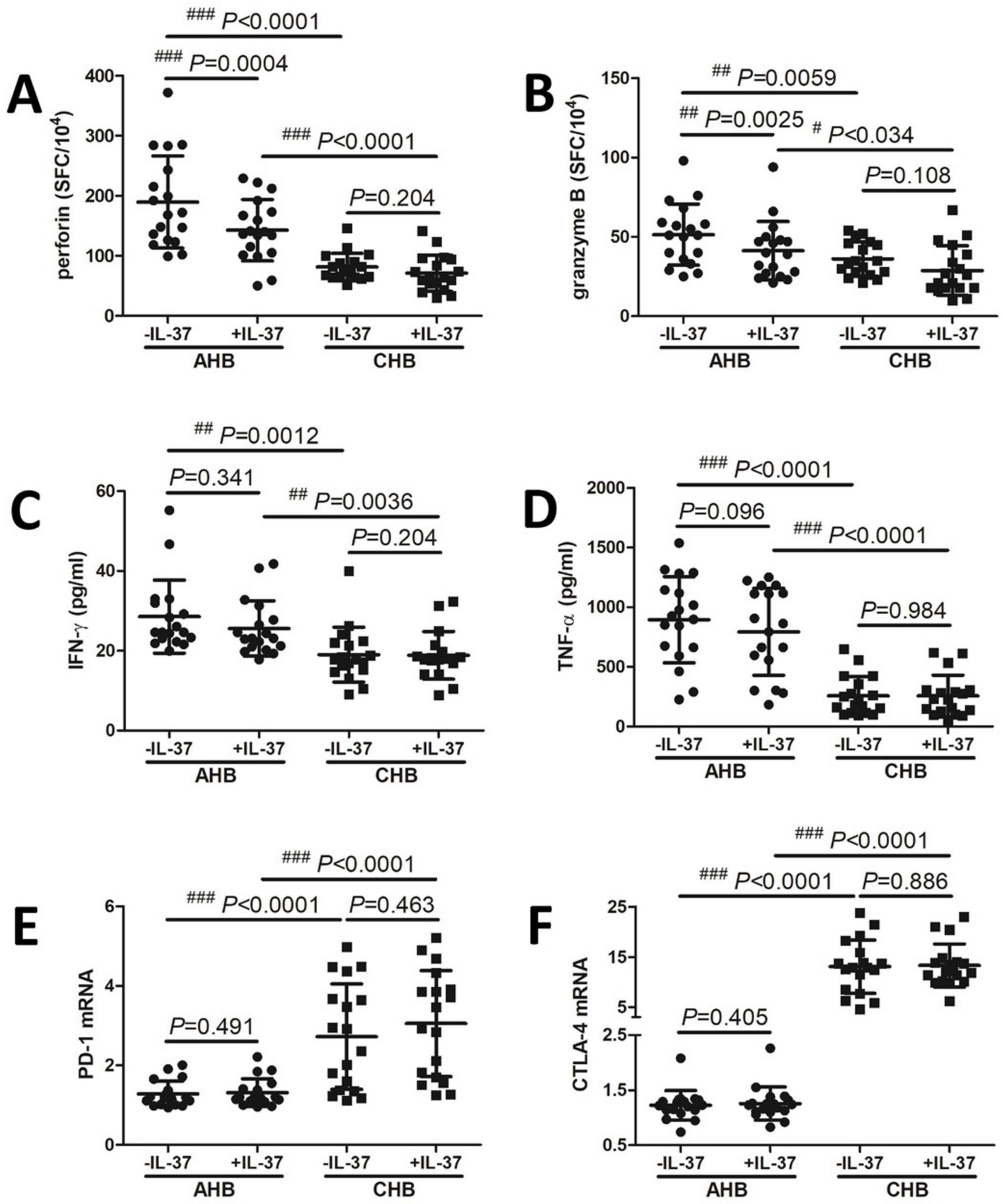
**The influence of recombinant human IL-37 to HBV peptides-induced production of perforin and granzyme B, secretion of interferon-γ (IFN-γ), and tumor necrosis factor-α (TNF-α), as well as programmed death-1 (PD-1) and cytotoxic T-lymophcyte associated protein-4 (CTLA-4) mRNA expression in CD8+ T cells in acute hepatitis B (AHB) and chronic hepatitis B (CHB) patients in vitro.** Purified CD8+ T cells from AHB patients (*n* ═ 18) and CHB patients (*n* ═ 18) were stimulated with HBV C genotype core peptide pool (2.5 µg/ml) in the presence or absence of recombinant human IL-37 (0.1 ng/ml) for 96 h. Perforin and granzyme B secretion was measured by enzyme-linked immunospot assay. (A) Perforin and (B) granzyme B secretion by CD8+ T cells was compared between cells with and without exogenous IL-37 stimulation in AHB and CHB patients. IFN-γ and TNF-α production was measured by enzyme-linked immunosorbent assay; (C) IFN-γ and (D) TNF-α production by CD8+ T cells was compared between cells with and without exogenous IL-37 stimulation in AHB and CHB patients. PD-1 and CTLA-4 mRNA expression in CD8+ T cells was measured by real-time polymerase chain reaction; (E) PD-1 and (F) CTLA-4 mRNA expression in CD8+ T cells was compared between cells with and without exogenous IL-37 stimulation in AHB and CHB patients. The Student’s *t* test and paired *t* test was used for comparison. IL-37: Interleukin-37; HBV: Hepatitis B virus; ^#^*P* < 0.05; ^##^*P* < 0.01; ^###^*P* < 0.001.

The influence of IL-37 to HBV peptides-induced secretion of perforin and granzyme B by CD8+ T cells as well as PD-1 and CTLA-4 mRNA expression in CD8+ T cells in AHB and CHB patients was investigated in vitro. Purified CD8+ T cells from 18 AHB patients and 18 CHB patients were stimulated with HBV C genotype core peptide pool in the presence or absence of recombinant human IL-37 for 96 h. Perforin and granzyme B secretion was measured by ELISPOT. HBV peptides-induced perforin and granzyme B secretion by CD8+ T cells was notably higher in AHB patients when compared with in CHB patients (Student’s *t* tests, *P* < 0.01, Figure 3A and 3B). Exogenous IL-37 stimulation inhibited perforin and granzyme B secretion by CD8+ T cells from AHB patients (paired *t* tests, *P* < 0.01, Figure 3A and 3B). However, there were no significant differences of either perforin or granzyme B secretion between CD8+ T cells with and without IL-37 stimulation in CHB patients (paired *t* tests, *P* > 0.05, Figure 3A and 3B). IFN-γ and TNF-α production was measured by ELISA. HBV peptides-induced IFN-γ and TNF-α production by CD8+ T cells was also significantly higher in AHB patients when compared with in CHB patients (Student’s *t* tests, *P* < 0.01, Figure 3C and 3D). FN-γ and TNF-α production by CD8+ T cells was comparable between cells with and without exogenous IL-37 stimulation in both AHB and CHB patients (paired *t* tests, *P* > 0.05, Figure 3C and 3D). PD-1 and CTLA-4 mRNA expression in CD8+ T cells was measured by real-time PCR. PD-1 and CTLA-4 mRNA expression in CD8+ T cells was robustly enhanced in CHB patients when compared with in AHB patients (Student’s *t* tests, *P* < 0.0001, Figure 3E and 3F). However, exogenous IL-37 stimulation did not affect either PD-1 or CTLA-4 mRNA expression in CD8+ T cells in both AHB and CHB patients (paired *t* tests, *P* > 0.05, Figure 3E and 3F).

### Exogenous IL-37 stimulation dampened HBV peptide-induced CD8+ T cell cytotoxicity in AHB patients

**Figure 4.**
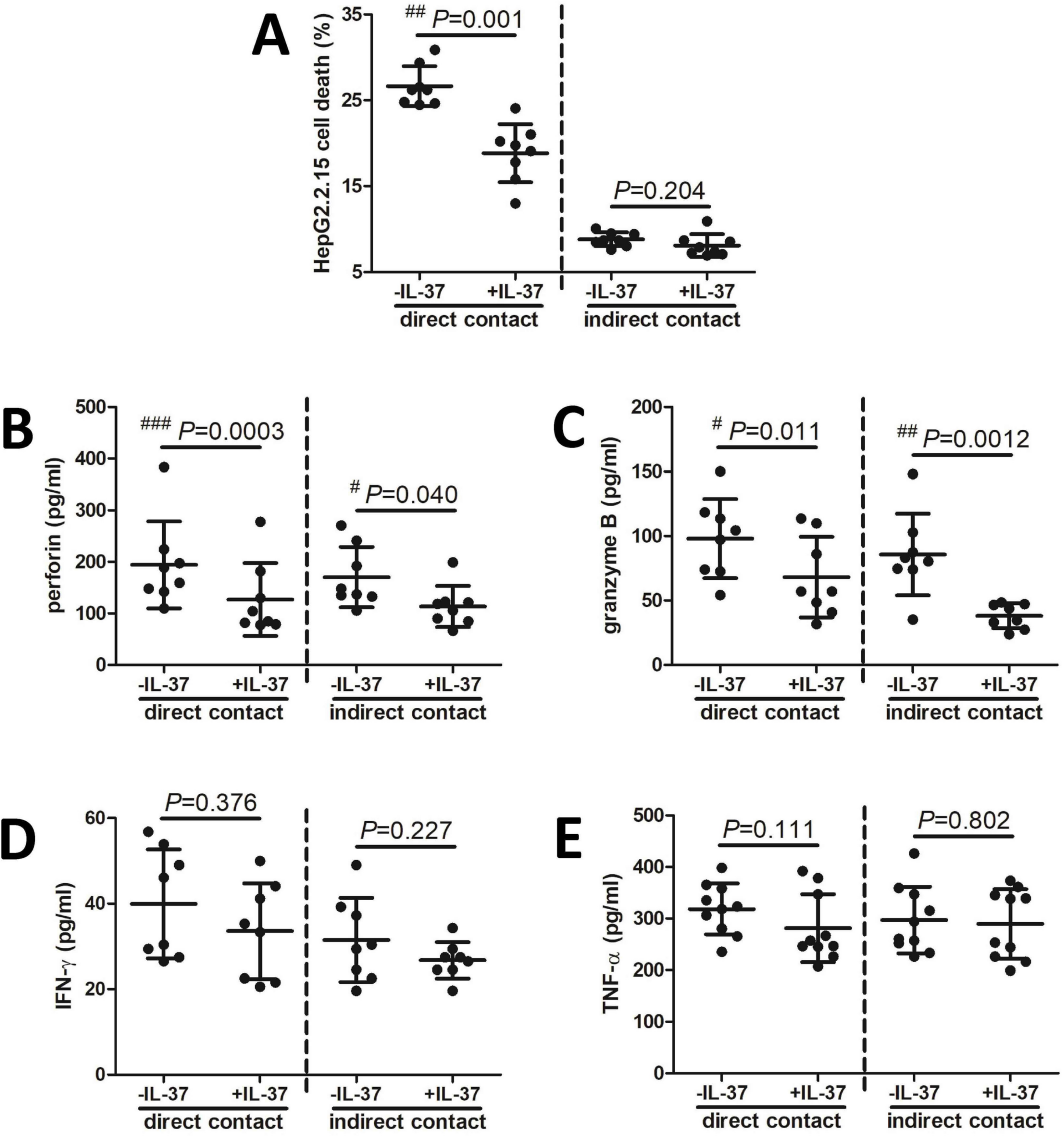
**The influence of recombinant human IL-37 to HBV peptide-induced CD8+ T cell cytotoxicity in acute hepatitis B (AHB) patients in vitro.** Purified CD8+ T cells from HLA-A*02 positive AHB patients (*n* ═ 8) were stimulated with recombinant human IL-37 (0.1 ng/ml) for 24 h and were cocultured with HepG2.2.15 cells in both direct contact and indirect contact manners in the presence of recombinant HBsAg (10 µg/ml) and HBc18-27 peptide (10 µg/ml). Supernatants were harvested 48 h post coculture. (A) CD8+ T cell-mediated HepG2.2.15 cell death was calculated by measuring LDH expression in the supernatants. The percentage of HepG2.2.15 cell death was compared between cells with and without exogenous IL-37 stimulation in direct contact and indirect contact coculture systems; (B) Perforin, (C) granzyme B, (D) interferon-γ (IFN-γ), and (E) tumor necrosis factor-α (TNF-α) levels in the cultured supernatants were measured by enzyme-linked immunosorbent assay, and were compared between cells with and without exogenous IL-37 stimulation in direct contact and indirect contact coculture systems. The paired *t* test was used for comparison; ^#^*P* < 0.05; ^##^*P* < 0.005; ^###^*P* < 0.0005.

The influence of IL-37 to HBV peptide-induced CD8+ T cell cytotoxicity in AHB patients was assessed in vitro. Purified CD8+ T cells from eight HLA-A*02 positive AHB patients were stimulated with recombinant human IL-37 for 24 h, and were cocultured with HepG2.2.15 cells in both direct contact and indirect contact manners in the presence of recombinant HBsAg and HBc18-27 peptide for 48 h. CD8+ T cell-mediated HepG2.2.15 cell death was calculated by measuring LDH expression in the cultured supernatants. Perforin, granzyme B, IFN-γ, and TNF-α levels in the cultured supernatants were also investigated. In direct contact coculture system, HBV peptide-induced CD8+ T cells-mediated HepG2.2.15 cell death was strongly prohibited by exogenous IL-37 stimulation (18.84 ± 3.37 % vs. 26.64 ± 2.32 %; paired *t* test, *P* ═ 0.001, Figure 4A). However, there was no significant difference of HBV peptide-induced CD8+ T cells-mediated HepG2.2.15 cell death between cells with and without IL-37 stimulation in indirect contact coculture system (8.08 ± 1.32 % vs. 8.81 ± 0.81 %, paired *t* test, *P* ═ 0.204, Figure 4A). Exogenous IL-37 stimulation suppressed perforin and granzyme B secretion in both direct contact and indirect contact systems (paired *t* tests, *P* > 0.05, Figure 4B and 4C). In contrast, there were no remarkably differences of either IFN-γ or TNF-α level in the supernatants between cells with and without IL-37 stimulation in both direct contact and indirect contact systems (paired *t* tests, *P* > 0.05, Figure 4D and 4E).

## Discussion

IL-37 has a potential ability to reduce excessive inflammation and to regulate innate and acquired immunity during both acute and chronic infections [20]. Severe acute respiratory syndrome coronaviurs (SARS-CoV-2) infection caused the elevation of plasma IL-37, which was associated with IL-6/IL-8 levels and benign clinical outcomes [21]. SARS-CoV-2 infection also induced IL-37 expression in inflammatory state, leading to subsequent suppression of IL-1β, TNF-α, and chemokines [22, 23]. In contrast, IL-37/SIGIRR axis was functionally compromised in human immunodeficiency virus (HIV)-infected individuals [24], although IL-37 expression was increased [24] and was associated with inflammation and the size of total viral reservoir in chronic HIV infection [25]. Importantly, serum IL-37 concentration was lower in patients with brucellosis than in healthy controls. In addition, IL-37 level was strongly reduced in acute brucellosis than in chronic brucellosis [26]. The natural history of HBV infection also contains acute and chronic hepatitis state. To the best of knowledge, we firstly investigated IL-37 and IL-37 receptor expression profile in AHB and CHB patients. We found that plasma IL-37 level was robustly down-regulated in AHB patients, but not in CHB patients. This was not consistent with previous reports regarding chronic HBV infection. Li and Meng et al. revealed that chronic active HBV infection induced elevated IL-37 expression and effective antiviral therapy reduced circulating IL-37 level in HBeAg seroconverted CHB patients [14, 27]. Serum IL-37 level was positively associated with ALT level in CHB patients [14, 27]. However, our current results indicated that reduced IL-37 was negatively correlated with liver inflammation, but was not related with HBV DNA in AHB patients. There might be several reasons contributed to the different results. Firstly, virus itself might not mediate IL-37 reduction because HBV DNA could be detectable in both AHB and CHB patients. Secondly, acute and chronic HBV infection presented different immune status. IL-37 expression might be attenuated in AHB patients since the requirement for proinflammatory mechanisms to eliminate HBV-infected hepatocytes. In contrast, IL-37 might suppress the liver damage of proinflammatory mechanisms in CHB patients. This process was similar to the status of acute and chronic brucellosis [26]. Thirdly, although we did not investigate transcription stage of IL-37 during HBV infection, it was shown that IL-37 mRNA expression was no change in either acute or chronic brucellosis [26]. Thus, the change in protein level of IL-37 might be regulated by post-translational mechanisms.

Vigorous, multifunctional viral specific CD8+ T cells play a central role in clearance of acute HBV infection and elimination of HBV-infected hepatocytes [28]. In contrast, CHB patients are characterized by dysfunctional or exhausted viral specific CD8+ T cell response, leading to persistent HBV infection [28–30]. The current data revealed robustly higher cytotoxic molecules and proinflammatory cytokines production as well as extremely lower immune checkpoint molecules expression in HBV peptides-induced CD8+ T cells from AHB patients compared with those from CHB patients, indicating the strong CD8+ T cell response in AHB patients, while weak CD8+ T cell activity in CHB patients. CD8+ T cells from HCs did not pre-expose to HBV antigens. Stimulation with HBV peptides did not induce immune response in controls. Due to the findings in the correlation between IL-37 and liver inflammation in AHB patients, the regulatory function of exogenous IL-37 to HBV peptides-induced CD8+ T cells was then assessed in vitro. Exogenous IL-37 stimulation only inhibited perforin and granzyme B production by HBV peptides-induced CD8+ T cells in AHB patients, but not in CHB patients. However, recombinant human IL-37 did not affect IFN-γ/TNF-α secretion as well as PD-1/CTLA-4 mRNA expressions in CD8+ T cells in both AHB and CHB patients. Although IL-37 was shown to decrease surface PD-1 expression in aged T cells in mouse [31] and inhibited sustained hepatic IFN-γ/TNF-α production in T cell-dependent liver injury [32], the current data still indicated that the immunosuppressive behavior of IL-37 to viral specific CD8+ T cells in AHB patients might be mainly through dampening cytotoxic molecules secretion. The differential regulatory function of IL-37 to CD8+ T cells in AHB and CHB patients might not be dependent on IL-37 receptor expression because both membrane-bound and soluble IL-37 receptor expressions were comparable between AHB and CHB patients. In our opinions, the following two reasons might contribute to the different regulatory function of IL-37 to CD8+ T cells in AHB and CHB patients. On the one hand, the immune status of AHB and CHB was completely different. The strong immune response in AHB patients and weak immune response in CHB patients might lead to the different responsiveness to IL-37 stimulation. On the other hand, we have demonstrated that circulating IL-37 level was reduced in AHB patients, but not in CHB patients. Thus, exogenous IL-37 stimulation might present more impressive modulatory activity to AHB patients. However, the mechanisms underlying IL-37 modulation to CD8+ T cells during HBV infection still need further elucidation.

The mechanisms of antigen specific CD8+ T cell-induced cytotoxicity involve direct cytolytic function to target cells and cytokine-mediated tissue damage [33]. The direct cytolytic activity of CD8+ T cells is mainly through perforin-granzyme signaling pathway, which was required direct cell-to-cell contact with target cells [34–36]. CD8+ T cells could also secrete proinflammatory cytokines, which also contribute to target cell death [34–36]. The in vitro direct contact and indirect contact coculture systems between effector CD8+ T cells and target cells are set up to assess the cytotolytic and cytokine-induced cytotoxicity independently [33–36]. HBV peptide-induced CD8+ T cells mediated extremely higher percentage of HepG2.2.15 cell death in direct contact coculture system than in indirect contact coculture system, indicating direct cell-to-cell contact dependent cytotoxicity played a predominate role in the induction of target cell death. IL-37 stimulation only suppressed CD8+ T cell-mediated HepG2.2.15 cell death in direct contact coculture, but not in indirect contact coculture system. This was consistent with the findings that IL-37 did not affect IFN-γ and TNF-α, which played essential role for CD8+ T cell cytotoxicity in indirect contact coculture system. Although exogenous IL-37 inhibited perforin and granzyme B secretion in both direct contact and indirect contact coculture, perforin–granzyme signaling required direct cell contact. These findings suggested that IL-37 might suppress hepatocytes injury and liver inflammation mainly through dampening cytolytic effector function of CD8+ T cells in AHB patients.

The limitation of the present study was lack of in vivo studies. The experimental evidence from animal research using HBV transgenic mouse model or HBV expressing plasmid hydrodynamic injected mouse model should be crucial for understanding the pathogenic mechanisms and direct effect to viral replication of IL-37 during HBV infection.

## Conclusion

In summary, our current data indicated that acute HBV infection-induced insufficient secretion of IL-37. Exogenous IL-37 suppressed HBV peptides-induced CD8+ T cell cytotoxicity mainly by inhibiting direct cytolytic activity. The comprehensive understanding of IL-37 modulation to CD8+ T cell may facilitate the controlling of liver inflammation during HBV infection.
